# Comparative transcriptomic and proteomic analyses provide insights into the key genes involved in high-altitude adaptation in the Tibetan pig

**DOI:** 10.1038/s41598-017-03976-3

**Published:** 2017-06-16

**Authors:** Bo Zhang, Yangzom Chamba, Peng Shang, Zhixiu Wang, Jun Ma, Liyuang Wang, Hao Zhang

**Affiliations:** 1 National Engineering Laboratory for Animal Breeding, China Agricultural University, No. 2 Yuanmingyuan West Rd., Beijing, 100193 China; 2 Tibet Agriculture and Animal Husbandry College, Linzhi, Tibet 860000 China

## Abstract

Tibetan pigs that inhabit the Tibetan Plateau exhibit striking phenotypic and physiological differences from lowland pigs, and have adapted well to extreme conditions. However, the mechanisms involved in regulating gene expression at high altitude in these animals are not fully understood. In this study, we obtained transcriptomic and proteomic data from the heart tissues of Tibetan and Yorkshire pigs raised in the highlands (TH and YH) and lowlands (TL and YL) via RNA-seq and iTRAQ (isobaric tags for relative and absolute quantitation) analyses, respectively. Comparative analyses of TH *vs*. YH, TH *vs*.TL, TL *vs*. YL, and YH *vs*. YL yielded 299, 169, 242, and 368 differentially expressed genes (DEGs), and 473, 297, 394, and 297 differentially expressed proteins (DEPs), respectively. By functional annotation of these DEGs and DEPs, genes that were enriched in the HIF-1 signaling pathway (*NPPA*, *ERK2*, *ENO3*, and *EGLN3*), VEGF signaling pathway (*ERK2*, *A2M*, *FGF1*, *CTGF*, and *DPP4*), and hypoxia-related processes (*CRYAB*, *EGLN3*, *TGFB2*, *DPP4*, and *ACE*) were identified as important candidate genes for high-altitude adaptation in the Tibetan pig. This study enhances our understanding of the molecular mechanisms involved in hypoxic adaptation in pigs, and furthers our understanding of human hypoxic diseases.

## Introduction

Low air pressure and low oxygen partial pressure at high altitude seriously affect the survival and development of human beings and other animals^1, 2^. Indigenous animals of the Tibetan Plateau exhibit heritable adaptations to this extreme environment due to natural selection; therefore, the Tibetan pig is an ideal animal model for research on the molecular ecology and pathology related to hypoxia^3–5^.

Adaptation to hypoxic conditions is a complex trait that is influenced by many factors^6^. Several genes exhibiting strongly selection have been identified in Tibetan populations by genome scanning, providing a possible genetic basis for adaptation to high-altitude conditions^7–9^. Likewise, a number of rapidly evolving, positively selected genes have been reported in the Tibetan pig^10^; however, the mechanisms involved in gene expression at high altitude are not fully understood. The development of RNA-seq and isobaric tags for relative and absolute quantitation (iTRAQ) technologies has enabled the identification of eukaryotic genes associated with complex traits via analysis of transcriptomic and proteomic profiles, with low bias, a large dynamic range, low frequency of false-positive signals, and high reproducibility. The iTRAQ method employs a set of amine-reactive isobaric tags to derivatize peptides at the N-terminus and at lysine side chains, thereby allowing for simultaneous protein identification and quantification via mass spectrometry analysis of peptide fragments (m/z range from 150 to 2,200) and signature ions (m/z values from 113 to 121), respectively. Both RNA-seq and iTRAQ have been widely used to screen for functional genes involved in muscle growth and lipid deposition in pigs and other domestic species^11–14^. An integrated analysis of the transcriptome and proteome of pig heart tissues at high and low altitudes would comprehensively profile hypoxic adaptations, and enable us to characterize the genes and proteins that are involved in this process.

Heart tissues were previously used to study hypoxic physiology and pathology^15, 16^. An increased heart rate, blood pressure, and other high-altitude responses result in changes in cardiac structure and function. In particular, animals populating the Tibetan Plateau exhibit strong cardiac function, and their cardiomyocytes show an ability to promote the expression of genes associated with adaptation to hypoxic conditions to protect cardiac cells and tissue structure, and increase blood circulation. Indeed, Tibetan pigs have adapted to high altitudes with well-developed hearts and lungs, and a blunted erythropoietic response^17^.

Cellular responses to hypoxia are reliant on controlled transcriptional and post-transcriptional events, in which certain genes are positively regulated and others either remain inactive or are actively repressed. In this study, we performed a comparative analysis of the transcriptomic and proteomic profiles of heart tissues obtained from Tibetan and Yorkshire pigs raised at high (TH and YH, respectively) and low (TL and YL, respectively) altitudes using RNA-seq and iTRAQ technologies. Via these analyses, we identified key genes and molecular mechanisms involved in the high- altitude adaptations of the Tibetan pig.

## Results

### Overview of RNA transcriptomic profiles of pig heart tissues

A total of 551 M paired-end reads were acquired from the RNA-seq experiment. Sequence alignment was performed using the pig genome (Sscrofa10.2) as a reference. Approximately 83.68% (82.07–85.18%) of the mapped reads were acquired from the RNA-seq experiment of which 76.26% (74.86–77.74%) were mapped to unique genomic locations. Additionally, of the total mapped reads, roughly 89% in each group corresponded to exons (Table S3, Fig. S1).

Gene expression levels were evaluated by counting the number of mapped reads per gene. In total, 18,585 expressed genes were detected in the heart tissues examined; of these, 15,701 were detected in each of the four groups (Fig. S2A). Less than 1% of the genes were expressed at greater than 1,000 FPKM (numbers of fragments per kilobase of exon per million mapped fragments); approximately 3% were expressed between 100 and 1,000 FPKM, and approximately 96% were expressed at less than 100 FPKM (Table S3, Fig. S2B). Expression value distributions were similar among the eight samples tested. Likewise, the FPKM values were evenly distributed amongst the four groups, and there were no outliers (Fig. S2C).

### Analysis of differentially expressed genes (DEGs)

Pair-wise comparisons with the strict criteria of |log_2_ (FC)| > 1 and *P* < 0.01 were utilized to detect DEGs (Fig. S3A). Specifically, 299 and 242 DEGs were detected in the comparisons of TH to YH (Table S4–1, Fig. S3B) and of TL to YL (Table S4–2, Fig. S3B), respectively, including 26 DEGs that were common to each comparison (Fig. S3C). Meanwhile, we detected 169 and 368 DEGs between TH and TL (Table S4-3, Fig. S3B) and YH and YL (Table S4-4, Fig. S3B), respectively, of which 24 overlapped (Fig. S3C).

### Functional analysis of DEGs

We used DAVID and Ingenuity Pathways Analysis (IPA), which have related capabilities but utilize different databases, to obtain a complete functional view of the DEGs. Using DAVID, we observed an overrepresentation of gene ontology (GO) terms related to “response to hormone stimulus” (19 genes, *P* = 3.15E-05) and “mitochondrion” (36 genes, *P* = 6.30E-05) in TH *vs*. YH, and “oxidation reduction” (51 genes, *P* = 1.77E-16) and “cofactor binding” (32 genes, *P* = 2.44E-15) in TL *vs*. YL (Table S5). We also observed an overrepresentation of the Kyoto Encyclopedia of Genes and Genomes (KEGG) pathways “ribosome” (15 genes, *P* = 8.52E-10) and “dilated cardiomyopathy” (7 genes, *P* = 9.08E-03) in TH *vs*. YH, and “glycolysis/gluconeogenesis” (9 genes, *P* = 3.02E-04) and “renin-angiotensin system” (5 genes, *P* = 1.22E-03) in TL *vs*. YL. Analysis of these two comparison groups also highlighted GOs and pathways, such as “regulation of blood pressure”, “vascular process in circulatory system”, “response to hypoxia”, and “complement and coagulation cascades pathway” (Table S5). Additionally, the mammalian target of rapamycin (mTOR) signaling pathway was detected in the TH *vs*. YH comparison; hypoxia-induced mTOR activation reduces mTOR targeting to enhance angiogenesis in response to hypoxia^18^. Moreover, we identified a variety of DEGs in pathways upstream of the mTOR pathway^19^, in the insulin signaling pathway (seven of the DEGs were designated to encode oxygen-independent regulators).

In the TH *vs*. TL analysis, the overrepresented KEGG pathways were “contractile fiber” (12 genes, *P* = 1.68E-08), “muscle contraction” (10 genes, *P* = 1.22E-03), “dilated cardiomyopathy” (6 genes, *P* = 5.35E-03), “complement and coagulation cascades” (5 genes, *P* = 1.01E-02), while in YH *vs*. YL, they were “oxidation reduction” (32 genes, *P* = 2.10E-10), “=2.10biosynthetic processl (12 genes, *P* = 7.72E-09), “glycolysis/gluconeogenesis” (9 genes, *P* = 1.37E-05), and “histidine metabolism” (6 genes, *P* = 1.79E-04). Notably, GOs and pathways, such as the “mitochondrion”^20^, “glucose metabolic process”, “dilated cardiomyopathy”^16^, and “cardiac muscle contraction”^21^ pathways, which play important roles in the hypoxia response, were also detected in TH *vs*. TL.

We also performed metabolic pathway analysis on the DEGs using IPA software. Based on the data obtained via this method, the main canonical pathways that were overrepresented in the TH *vs*. YH and TL *vs*. YL comparisons were related to hepatic fibrosis/hepatic stellate cell activation (*FN1*, *CTGF*, *CCL5*, *A2M*, and *FGF1*, and *COL1A2*, *CTGF*, *AGT*, and *COL3A1*; *P* = 3.85E-02 and *P* = 2.58E-02, respectively), and glucocorticoid receptor signaling in TH *vs*. YH (*DUSP1*, *SLPI*, *CCL5*, and *A2M*, and *DUSP1* and *AGT*; *P* = 1.82E-01 and *P* = 3.66E-01, respectively). Thus, depending on the environment, Tibetan pigs modulated the expression of distinct DEGs to adapt to hypoxic conditions. In addition, interesting pathways such as the hypoxia-inducible factor 1 (HIF-1) signaling pathway (*EGLN3*) were detected in both the TH *vs*. YH and TH *vs*. TL comparisons (Table S6).

Of the primary molecular and cellular functions that were significantly overrepresented in the four comparison groups, according to IPA, the most relevant were involved in “cardiovascular system development and function” in TH *vs*. YH (23 genes, *P* = 1.33E-09–2.40E-03), “cardiac hypertrophy” in TL *vs*. YL (5 genes, *P* = 1.393E-05–1.90E-01), “cardiovascular system development and function” in TH *vs*. TL (16 genes, *P* = 4.22E-07–4.69E-03), and “skeletal and muscular system development and function” in YH *vs*. YL (3 genes, *P* = 7.05E-06–9.10E-03).

Among the related specific functions of heart tissue, the main functions identified by IPA in TH *vs*. YH were “angiogenesis” (*ANXA2*, *CCL5*, *CD151*, *CD44*, *CTGF*, *EGLN3*, *FGF1*, *FN1*, *HDAC9*, *ITGA5*, *JUNB*, *NR4A1*, *PFKM*, *SLPI*, and *TGM2*; *P* = 1.33E-09), “binding of endothelial cells” (*ANXA2*, *CCL5*, *CD151*, *CD44*, *FGF1*, *FN1*, and *NR4A1*; *P* = 7.07E-09), and “development of blood vessel” (*ANXA2*, *ATF3*, *CCL5*, *CD151*, *CD44*, *CTGF*, *EGLN3*, *FGF1*, *FN1*, *HDAC9*, *ITGA5*, *JUNB*, *NR4A1*, and *TGM2*; *P* = 9.53E-08); in TL *vs*. YL were “hypertrophy of cardiomyocytes” (*AGT*, *CRYAB*, *CTGF*, and *DUSP1*; *P* = 1.39E-05) and “hypertrophy of heart” (*AGT*, *CRYAB*, *CTGF*, and *DUSP1*; *P* = 2.44E-04); in TH *vs*. TL were “formation of endothelial tube” (*ATF3*, *CCL2*, *CD44*, *FN1*, and *PPP1R14B*; *P* = 4.22E-07) and “angiogenesis” (*CCL2*, *CD151*, *CD44*, *CRYAB*, *FGF1*, *FN1*, *HSPB8*, *ITGAV*, *JUNB*, *PFKM*, and *TGM2*; *P* = 5.22E-06); and in YH *vs*. YL were “quantity of muscle cells” (*AGT*, *CCL2*, and *FLNC*; *P* = 7.05E-06) and “quantity of smooth muscle cells” (*AGT* and *CCL2*; *P* = 1.03E-04) (Table S6). Notably, we obtained the same pathways via functional analysis of TH *vs*. TL and YH *vs*. YL by IPA. Thus, even when inhabiting the same environment, Tibetan and Yorkshire pigs utilize distinct genes to adapt to hypoxia. All of the DEGs that were related to hypoxic adaptation in the four groups are shown in Table 1.

**Table 1:** Potential key differentially expressed genes (DEGs) and their functions related to hypoxic adaption in the Tibetan pig.

Gene	TH/YH	TL/YL	TH/TL	YH/YL	Functional analysis
*A2M*	0.44	0.25	1.43	0.81	Complement and coagulation cascades, blood microparticle, VEGF signaling pathway
*AGT*	0.63	2.94	0.57	2.68	Renin-angiotensin system, vasculature development, muscle contraction, blood vessel development, regulation of blood pressure
*ANXA2*	3.44	1.18	4.19	1.44	angiogenesis, vasculature development,
*ATF3*	6.18	17.30	0.27	0.77	gluconeogenesis, DNA binding
*CD44*	2.82	0.82	5.80	1.68	ECM-receptor interaction, Hematopoietic cell lineage
*CCL5*	0.48	1.11	0.75	1.71	immune response, response to insulin stimulus
*COL3A1*	1.58	4.95	0.50	1.55	response to radiation, blood vessel development
*CRYAB*	2.20	0.68	4.68	1.45	mitochondrion, response to hypoxia, response to reactive oxygen species
*CTGF*	5.72	3.31	1.58	0.91	lung development, blood vessel development, vasculature development, angiogenesis, blood vessel morphogenesis, reactive oxygen species metabolic process, cardiovascular development, VEGF signaling pathway
*DUSP1*	3.92	2.96	1.50	1.13	MAPK signaling pathway
*EGLN3*	0.47	0.72	0.78	1.21	Pathways in cancer, renal cell carcinoma, oxidation-reduction process, response to hypoxia, HIF-1 signaling pathway
*FGF1*	4.32	1.55	0.33	1.59	MAPK signaling pathway, Pathways in cancer, blood vessel morphogenesis, blood vessel development, respiratory system development, cardiovascular development, VEGF signaling pathway
*DPP4*	0.22	0.31	0.55	0.75	response to hypoxia, response to oxygen levels, VEGF signaling pathway
*HBB*	2.27	1.94	2.00	1.71	cardiac myofibril assembly, hemoglobin complex, oxygen transport
*HBA*	2.26	2.73	1.60	1.93	cardiac myofibril assembly, hemoglobin complex, oxygen transport
*NPPA*	37.60	2.31	27.27	1.68	regulation of blood vessel size, cardiac muscle hypertrophy in response to stress, HIF-1 signaling pathway, regulation of blood vessel size, circulatory system process, regulation of blood pressure, hypertrophy in response to stress
*NPPB*	41.96	0.17	138.23	0.57	regulation of blood vessel size, regulation of blood vessel size, circulatory system process
*DECR1*	0.42	1.02	0.40	0.97	Mitochondrion, reductase (NADPH) activity, NADPH binding
*TGFB2*	9.24	1.74	2.03	0.38	heart development, vasculature development, cardiac muscle tissue development, response to oxygen levels, response to hypoxia, angiogenesis, regulation of heart contraction, immune system development, pathways in cancer, TGF-beta signaling pathway
*PDLIM3*	2.03	2.58	4.33	5.49	heart development, cardiovascular development
*FOS*	14.93	24.97	0.54	0.91	DNA binding, cellular response to reactive oxygen species, pertussis, pathways in cancer

TH, Tibetan highland pig; TL, Tibetan lowland pig; YH, Yorkshire highland pig; YL, Yorkshire lowland pig.

Based on functional annotation, 21 DEGs were associated with hypoxia (Table 1), which involved the GO terms of “response to hypoxia”, “blood vessel development”, “response to reactive oxygen species”, “circulatory system process”, “regulation of heart contraction”, and “immune system development”, as well as the HIF-1 signaling pathway, the mitogen-activated protein kinase (MAPK) signaling pathway, pathways in cancer, glycolysis/gluconeogenesis, and the TGF-beta signaling pathway. Three genes, *NPPA* (natriuretic peptide type A, also known as *ANP*), *NPPB* (natriuretic peptide type B, also known as *BNP*), and *FOS* (FBJ murine osteosarcoma viral oncogene homolog), exhibited substantial differential expression, with fold changes greater than 14 in TH *vs*. YH (Table 1). In particular, the expression level of *NPPB* in TH was 41.96, which was 138.23-fold higher than that in YH and TL, and may therefore exert a positive effect on hypoxic adaptation in these animals. Similarly, *NPPA* was also highly expressed in this group, with an expression level of 37.60, which was 27.27-fold higher than that in YH and TL. In humans, *ANP* and *BNP* constitute the dual natriuretic peptide system of the heart. Numerous studies have clearly shown that hypoxia stimulates atrial natriuretic peptide (ANP) secretion, resulting in cellular adaptation to hypoxia and protection of the ischemic heart^22–24^.

### Overview of protein identification and quantification by iTRAQ

A total of 128,390 spectra were obtained from the 8PLEX liquid chromatography tandem-mass spectrometry (LC-MS/MS) analysis (Fig. S4A). Pooling of the samples from both groups resulted in identification of 12,880 peptides at the peptide level, including 2578 proteins that had been originally identified with false discovery rates (FDR) ≤0.01, and with high confidence of the correct peptide sequence assignment. The numbers of proteins identified at distinct molecular weight ranges were as follows: 10–20 kDa (433), 20–30 kDa (443), 30–40 kDa (370), 40–50 kDa (318), 50–60 kDa (246), and 60–70 kDa (138). In total, these proteins accounted for 75.56% of those identified (Fig. S4B). In addition, the majority of the proteins were identified with high peptide coverage; 95.30% of the proteins had less than 50% sequence coverage, while 4.70% had greater than 50% sequence coverage (Fig. S4C). Among the proteins identified, 64.08% were represented by fewer than five peptides (Fig. S4D), indicating good sequence coverage of the proteins identified.

### Analysis of differentially expressed proteins (DEPs)

**Figure 1:**
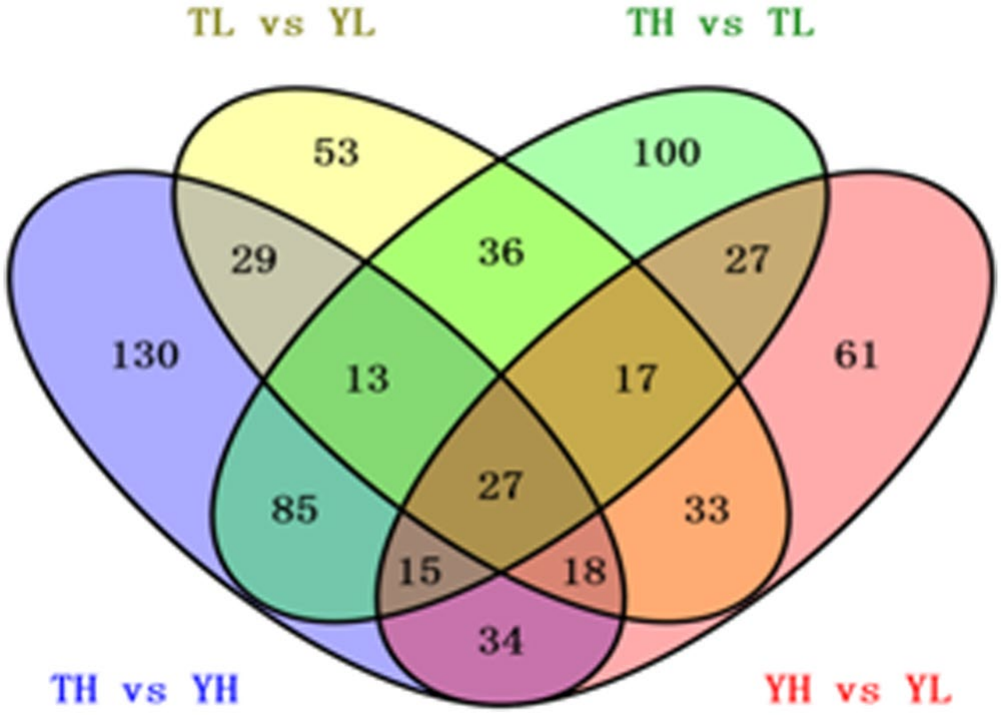
Venn diagram of differentially expressed genes (DEGs) among the four comparison groups. TH, Tibetan highland pig; TL, Tibetan lowland pig; YH, Yorkshire highland pig; YL, Yorkshire lowland pig.

Based on the selection criteria of FC > 1.2 or <0.83, we detected 473, 297, 394, and 297 DEPs in the TH *vs*. YH, TL *vs*. YL, TH *vs*. TL, and YH *vs*. YL comparisons, respectively. Specifically, 253 up-regulated and 220 down-regulated DEPs were identified in the TH *vs*. YH comparison, while 115 and 182, 275 and 119, and 179 and 188 up-regulated and down-regulated proteins were detected in TL *vs*. YL, TH *vs*. TL, and YH *vs*. YL, respectively (Table S7, Fig. S5). Notably, there were 87 and 86 overlapping proteins in the TH *vs*. YH and TL *vs*. YL, and in TH *vs*. TL and YH *vs*. YL comparisons, respectively (Table S8, Fig. 1).

### Validation of DEGs and DEPs

To validate the accuracy of the DEGs detected by RNA-seq analysis, we used real-time reverse transcription-quantitative polymerase chain reaction (RT-qPCR) to evaluate the expression levels of eight DEGs: *ADHFE1*, *CCL5*, *CKM*, *DECR1*, *DPP4*, *EGLN3*, *FOS*, and *SLA-5*. The expression levels of these genes in each group are shown in Fig. S6. The eight genes selected were differentially expressed among the four comparison groups (TH *vs*. YH, TH *vs*.TL, YH *vs*. YL, and TL *vs*. YL), and the RNA-seq data were concordant with those obtained by RT-qPCR. Moreover, RT-qPCR analysis confirmed the DEG expression patterns observed in the two pig breeds at each altitude (Fig. S7).

Notably, the results of previous studies suggest that the label-free method is typically more efficient, in regard to the number of proteins identified, than the iTRAQ approach^25–27^. In the current study, a total of 238 of the 351 (67.81%) and 686 (34.69%) proteins identified using the iTRAQ and label-free methods, respectively, were identified by both methods (Fig. S8A). For these proteins, the fold changes observed via each method were plotted, and a correlation with an R^2^ value of 0.604 was calculated (Fig. S8B). Of the overlapping 238 proteins, only three were found to be differentially expressed in divergent directions (i.e., up-regulation *vs*. down-regulation) in the comparison of TH *vs*. YH via iTRAQ and label-free experiments. Indeed, the overall trends of the commonly quantified proteins were in agreement between the two methods.

### Functional analysis of DEPs

**Figure 2:**
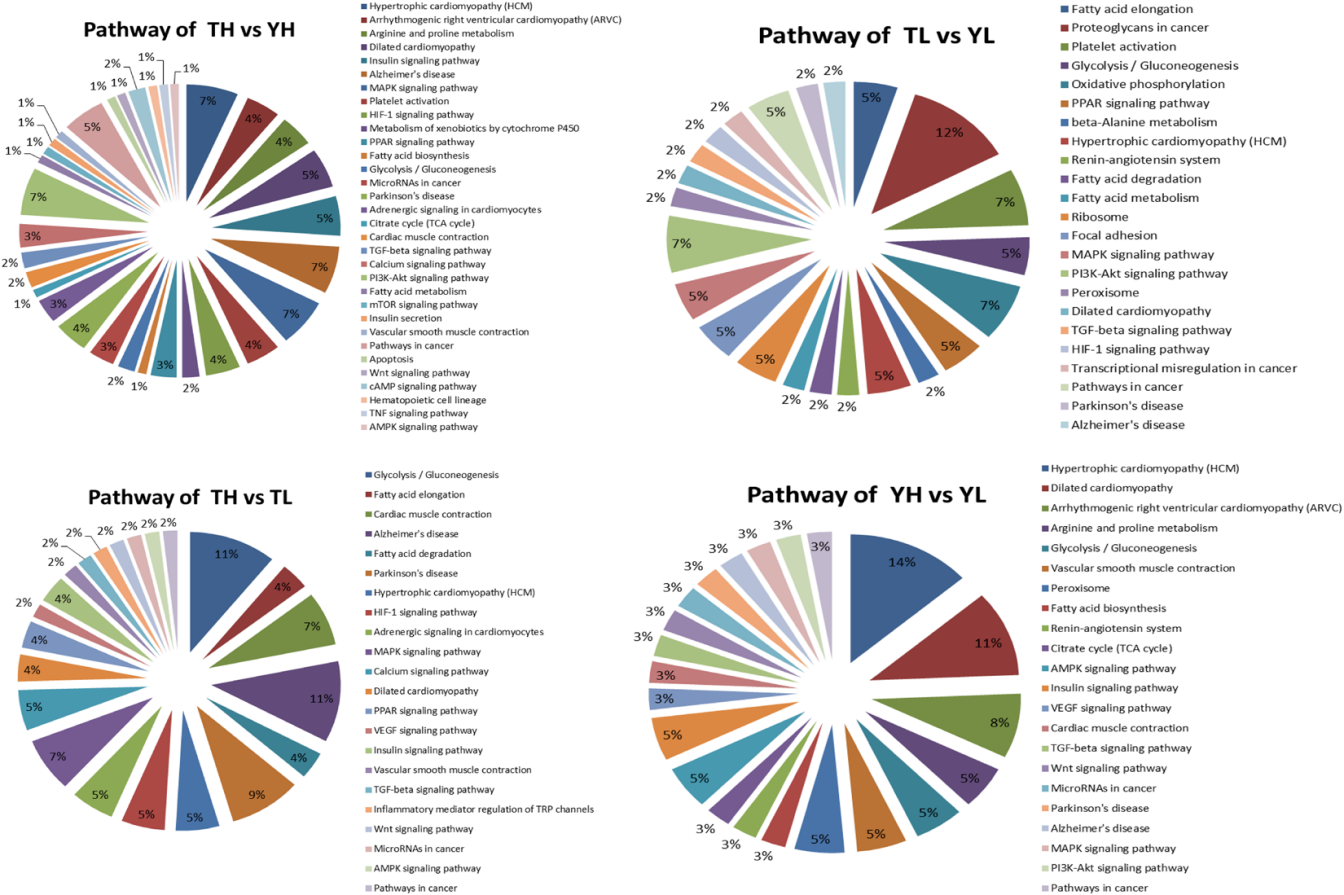
Differentially expressed protein (DEP) pathway analysis. TH, Tibetan highland pig; TL, Tibetan lowland pig; YH, Yorkshire highland pig; YL, Yorkshire lowland pig.

To better understand the biological functions of DEPs in Tibetan and Yorkshire pigs at different altitudes, the DEPs were further classified to identify pathways in the heart tissues based on GO and KEGG functional annotations (Table S9, Fig. 2). In TH *vs*. YH and TL *vs*. YL, the GO terms were mainly associated with “mitochondrion” (involved in 39 proteins and 44 proteins, respectively), “oxidation reduction” (involved in 23 proteins and 23 proteins, respectively), “blood circulation” (contained proteins such as CAV2, ACE, RENBP, AGT, MYL1, COL1A2, EPHX2, ERAP1, STAT1, HBB, TCAP, and MYH6), “NAD or NADH binding” (involved in 5 proteins and 4 proteins, respectively), “response to oxygen levels” (involved in 8 proteins and 7 proteins, respectively), and “iron ion homeostasis” (contained proteins such as TF, TFRC, HPX, FTH1, HP, and CP). In addition, we found interesting GOs in TH *vs*. YH, such as “gas transport” (*HBQ1*, *CA2*, *AQP1*, and *HBB*), “regulation of blood pressure” (*ACE*, *RENBP*, *AGT*, *COL1A2*, *EPHX2*, *ERAP1*, and *HBB*), and “oxygen and reactive oxygen species metabolic process” (*CRYAB*, *AGT*, *EPHX2*, *BNIP3*, and *PARK7*).

It is worth noting that 2–5% of the DEPs detected in the TH *vs*. YH, TL *vs*. YL, and TH *vs*.TL comparisons belonged to the HIF-1 signaling pathway. Likewise, 3–5% of the DEPs identified in the in TH *vs*. TL and YH *vs*. YL comparisons were associated with the vascular endothelial growth factor (VEGF) signaling pathway, which regulates angiogenesis and plays important roles in adaptation to hypoxic conditions. Meanwhile, the MAPK signaling pathway was enriched in 3–7% of the DEPs and pathways in cancer were enriched for 3–5% of the DEPs in the four comparison groups. The MAPK signaling pathway participates in HIF activation, and is involved in various cellular functions, including cell proliferation, differentiation, and migration^28^, while the proteins in pathways involved in cancer influence sustained angiogenesis, cellular proliferation, genomic damage, and inhibition of differentiation, all of which are closely related to cancer growth and development under hypoxic conditions^29^. In addition, the insulin signaling pathway, hypertrophic cardiomyopathy (HCM), glycolysis/gluconeogenesis, and dilated cardiomyopathy can be involved in hypoxic adaptation. All of the potential DEPs that were related to hypoxic adaptation in the four groups are shown in Table 2.

**Table 2:** Summary of hypoxia-regulated differentially expressed proteins (DEPs).

Protein	TH/YH	TL/YL	TH/TL	YH/YL	Functional analysis
MYL7	2.05	0.79	1.60	0.62	muscle contraction, actin cytoskeleton, actin binding, ATPase activity, contractile fiber part
PTPMT1	1.46	0.67	1.80	0.83	mitochondrion, inflammatory response, acute inflammatory response
PDLIM3	1.60	1.21	1.34	1.02	heart development, actin cytoskeleton, contractile fiber, cardiovascular development
A2M	1.42	0.72	1.62	0.83	Complement and coagulation cascades, blood microparticle, inflammatory response, regulation of immune effector process, VEGF signaling pathway
C6	1.29	0.78	1.28	0.77	inflammatory response, positive regulation of immune system process, acute inflammatory response, positive regulation of immune response, response mediated by circulating, defense response
CRYAB	1.46	0.86	1.41	0.82	response to hypoxia, mitochondrion, response to reactive oxygen species, oxygen and reactive oxygen species metabolic process, muscle contraction, oxygen species metabolic process
PDK4	1.10	1.02	1.45	1.34	mitochondrion, ATP binding, mitochondrion, reactive oxygen species metabolic process, glucose metabolic process
ERK2	1.20	0.98	1.15	0.94	HIF-1 signaling pathway, mTOR signaling pathway, TGF-beta signaling pathway, Vascular smooth muscle contraction, VEGF signaling pathway, cardiac muscle contraction
ACE	1.25	0.77	1.30	0.80	Vasculature development, response to oxygen levels, response to hypoxia, immune system development, regulation of blood pressure, heart contraction, Renin-angiotensin system, blood circulation
NAPG	1.24	0.77	1.21	0.75	mitochondrion, inflammatory response, actin filament binding,
CPS1	1.22	0.44	1.49	0.54	mitochondrion, glucose metabolic process, oxidation of organic compounds
HSPE1	0.98	1.50	0.80	1.23	inflammatory response, mitochondrion
DECR1	0.95	1.59	0.81	1.35	mitochondrion, 2, 4-dienoyl-CoA reductase (NADPH) activity, NADPH binding, oxidation reduction, iron ion homeostasis, muscle contraction
AHSG	0.72	1.20	0.78	1.30	inflammatory response, defense response,
ENO3	0.76	1.20	0.65	1.03	HIF-1 signaling pathway, glycolysis/gluconeogenesis, glucose metabolic process
MRPS26	0.63	1.40	0.68	1.52	mitochondrion, ribosome
DCN	0.50	0.80	0.80	1.29	TGF-beta signaling pathway
COL3A1	0.41	0.70	0.83	1.41	response to radiation, blood vessel development, Platelet activation
COL1A2	0.40	0.80	0.83	1.65	blood circulation, regulation of blood pressure, small GTPase mediated signal transduction
KRT8	0.32	0.67	0.75	1.56	contractile fiber part

TH, Tibetan highland pig; TL, Tibetan lowland pig; YH, Yorkshire highland pig; YL, Yorkshire lowland pig.

### Integrated analysis of DEGs and DEPs and iTRAQ and RNA-seq data

In total, 18,585 genes and 2,578 proteins were identified in the RNA-seq and iTRAQ analyses, of which 10,001 and 2,170 were annotated, respectively. Using these methods, the expression of 1,402 genes was detected at both the mRNA and protein levels (Fig. S9A). The proteome and transcriptome reflect gene expression at two different levels, and one of the goals of the combined analysis was to achieve data complementation and to obtain more complete information regarding the gene expression profiles of each organism at high and low altitudes. We obtained 473, 297, 394, and 297 DEPs in TH *vs*. YH, TL *vs*. YL, TH *vs*. TL, and YH *vs*. YL, respectively, of which 378, 220, 319, and 226, respectively, were annotated. A total of 26, 8, 23, and 7 genes were identified as DEGs and DEPs in the TH *vs*. YH, TL *vs*. YL, TH *vs*. TL, and YH *vs*. YL comparisons, respectively (Fig. S9B). The Pearson correlation coefficients obtained from a log_2_ function for TH *vs*. YH, TL *vs*. YL, TH *vs*. TL, and YH *vs*. YL were 0.40, 0.47, 0.60, and 0.42, respectively (Fig. S9C), indicating that only partial correlations were found at the mRNA and protein levels of overall gene expression. In summary, based on these results, we suggest that a substantial degree of post-transcriptional regulatory activity occurs during hypoxic adaptation, which has not been described in previous studies that focused solely on analyses of transcriptional and/or translational regulation.

## Discussion

The Tibetan pig, which inhabits high-altitude regions on the Qinghai-Tibet Plateau, has evolved genetic adaptations to these extreme elevations via natural and artificial selection^3^. Previous studies demonstrated that Tibetan pigs have a distinct suite of phenotypic and physiological characteristics, in regard to pulmonary vascular structures and blood flow^30, 31^. Sequencing of multiple individuals from various pig breeds revealed that certain genomic regions, including genes involved in the hypoxia response, olfaction, energy metabolism, and drug responses, are under selection in the Tibetan pig^10^. Moreover, miRNA-seq analysis identified several miRNAs that play regulatory roles in the hypoxic adaptation of these animals^4^. High-altitude adaptation is not controlled by a single gene, but by multiple evolved genetic adaptations acting in concert^32^. In the current study, we screened for key genes and proteins related to hypoxic adaptation by comparing Tibetan and Yorkshire pigs raised at different altitudes via RNA-seq and iTRAQ protein sequencing analyses. Subsequent functional enrichment analysis of each DEG and DEP identified via these analyses using GO and KEGG software identified 21 DEGs (*A2M*, *AGT*, *ANXA2*, *ATF3*, *CD44*, *CCL5*, *COL3A1*, *CRYAB*, *CTGF*, *DUSP1*, *EGLN3*, *FGF1*, *DPP4*, *HBB*, *HBA*, *NPPA*, *NPPB*, *DECR1*, *TGFB2*, *PDLIM3*, and *FOS*) and 20 DEPs (MYL7, PTPMT1, PDLIM3, A2M, C6, CRYAB, PDK4, ERK2, ACE, NAPG, CPS1, HSPE1, DECR1, AHSG, ENO3, MRPS26, DCN, COL3A1, COL1A2, and KRT8) that appear to be associated with adaptation to hypoxic conditions in the Tibetan pig. In particular, A2M, COL3A1, CRYAB, DECR1, and PDLIM3 were identified as both DEGs and DEPs.

Under hypoxic conditions, VEGF-mediated induction of *MEF2C* and its target gene *A2M* is strongly reduced, as is the expression of *HLX* and *UNC5B*. Together, these data suggest that up-regulation of *MEF2C*/*A2M* (and *HLX*/*UNC5B*) by VEGF is most strongly pronounced under normoxic conditions, and is inversely correlated with hypoxia^33^. We found that the expression level of *A2M* in TH and TL hearts was higher than that in YH and YL hearts, respectively, which could explain why Tibetan pigs adapt better to hypoxic conditions than Yorkshire pigs, regardless of altitude. Meanwhile, TH hearts exhibited 4.68-fold higher levels of CRYAB expression than YH and TL hearts. Given that the siRNA mitochondrial pathway is activated in hypoxic cardiomyocytes in the presence of CRYAB, as evidenced by increased production of cytosolic cytochrome c^34^, our findings indicate that enhanced expression of this protein could comprise another reason for the superior adaption to hypoxic conditions of TH, compared to YH or TL. PDLIM3 (PDZ and LIM domain protein 3, also known as ALP) is necessary to maintain normal myocardial contractility. GO annotations related to this gene include sion. In summary, based nding and structural constituent of muscle”, and cytoskeletal mutations are known causes of genetically based forms of dilated cardiomyopathy. Moreover, the disruption of the gene encoding ALP is associated with right ventricular chamber dilation and dysfunction, directly implicating α-actinin-associated proteins in the onset of cardiomyopathy^35^. PDLIM3-specific up-regulation in Tibetan pigs might therefore increase cardiovascular permeability, promote vascular endothelial cell proliferation and migration, and increase the regulation of cardiovascular development to prevent the occurrence of cardiomyopathy.

In this study, we identified a set of associated DEGs (*CD44*, *CTGF*, *DPP4*, *CRYAB*, *EGLN3*, *FGF1*, and *FOS*) with at least one functional enrichment, such as “angiogenesis”, “binding of endothelial cells”, “development of blood vessel”, MAPK signaling, HCM, and pathways in cancer (Table 1). Of these, *CTGF* (connective tissue growth factor) and *FGF1* (fibroblast growth factor 1), which are involved in blood vessel and vascular development, were expressed at significantly higher levels in TH than in YH, suggesting an important role for the cardiovascular system in hypoxia adaptation. In particular, CTCF restricts upstream enhancers from activating VEGF, thereby inhibiting the induction of VEGF and angiogenesis^36^. Hypoxia is the principal driver of induction of VEGF during both physiological and pathological angiogenesis^37^. *FGF1* is recognized as an angiogenesis factor *in vivo*, and angiogenesis is known to be induced by hypoxia^38^. Our findings therefore indicate that Tibetan pigs must increase *CTGF* and *FGF1* expression and modify their cardiovascular response to hypoxia by increasing blood flow and cardiac pumping.

The expression levels of *CD44* and *PDLIM3* were also significantly higher in the heart tissues of TH than in those of YH, TL, or YL. CD44 (CD44 molecule) transmembrane glycoproteins are cell adhesion molecules associated with aggressiveness and metastasis^37–40^, and cycling hypoxia has been shown to increase the population of CD44^+^/CD24^−^ cells in a metastatic breast cancer cell line^41^. Therefore, it seems probable that *CD44* expression is regulated by hypoxia. The functional effects of the up-regulation of *CD44* and its variant isoforms under hypoxic conditions, particularly in the context of hyaluronan levels, should be considered, as cell signaling events that promote anchorage-independent tumor cell growth, survival, migration, and metastasis occur through the binding of hyaluronan with CD44^40, 42, 43^. In addition, expression of *FOS* (also known as *c-FOS*), which was previously reported to be associated with the hypoxia response^44, 45^, was 17.3-fold higher in TL than in YL. Together, these data suggest that characterization of DEGs that differ between the two pig breeds could provide clues to understanding the distinct hypoxic adaptations of these animals.

According to the GO and pathway analyses, the DEGs *CRYAB* (crystallin, alpha B), *DPP4* (dipeptidyl-peptidase 4), and *EGLN3* (egl nine homolog 3) comprise three functional candidate genes that were related to “response to hypoxia”. Indeed, *EGLN1* and *EGLN3* are strongly induced by hypoxia in most cell types^46^. his effect is likely important for cellular adaptation to hypoxic conditions, and is responsible for the increased oxygen-mediated HIF-1a degradation observed after long periods of hypoxia^47^. Indeed, the induction of *EGLN3* mRNA expression by hypoxia is particularly significant when compared to that of other hypoxia-responsive genes^44, 46, 48, 49^. *DPP4* is a novel marker of HIF-1 activity in tumors^50^. In our study, the *DPP4* expression level in TH hearts was 0.22-fold higher than that in YH hearts, and 0.31-fold higher than in TL or YL hearts.

In the presence of low atmospheric oxygen, the oxygen levels in highland Yorkshire pigs introduced from the mainland were correspondingly low, and the difference in oxygen levels between capillary blood and mitochondria was small, resulting in hypoxia. A series of genes, such as *FLT-1* [fms-related tyrosine kinase 1 (vascular endothelial growth factor/vascular permeability factor receptor)], which was categorized with the GO term “vascular regulation” and HIF-1 signaling pathway in the YH group and is considered the strongest of the VEGF receptors, as well as the HIF-1 signaling pathway, were up-regulated in Yorkshire pigs in responses to hypoxia in the YH group. Hypoxic exercise can result in increased production of VEGF and Flt-1 in muscle tissue; following VEGF protein production, Flt-1 expression at the vascular endothelial cell membrane can be regulated via autocrine or paracrine responses and participate in muscle tissue angiogenesis^51^. In this study, expression of the Flt-1 gene was the highest in YH and the lowest in TH, possibly because compensatory responses increase vascular permeability in response to a hypoxic environment.

The specific KEGG enrichments in the Yorkshire pig were primarily concentrated in energy metabolism pathways, such as the glycolysis pathway. This was not surprising as, under hypoxic conditions, myocardial contraction requires increased adenosine triphosphate (ATP) consumption. However, while most differentiated cells employ mitochondrial oxidative phosphorylation to generate ATP to maintain cell processes under normoxic conditions, cells exposed to hypoxia typically produce ATP via glycolysis, which produces lower levels of this energy source than aerobic oxidation. As such, under hypoxic conditions, animals must up-regulate glycolysis to meet the consumption requirements of myocardial cells. In particular, the primary DEGs associated with energy metabolism in Yorkshire pigs were *ITGA5*, *PCK1*, and *PCK2*. Glycolysis produces phosphoenolpyruvate (PEP), and the overexpression of *PCK1* can lead to increased PEP production and gluconeogenesis^52^.

In this study, many genes were found to exhibit consistency in regard to changes in transcript and protein levels; however, there were several cases in which inconsistent results were obtained by RNA-seq and iTRAQ analysis, suggesting that post-transcriptional regulation plays an important role in the adaptive response to hypoxia in Tibetan pigs. Both transcriptomic and proteomic data are important for deciphering the molecular processes involved in this process. Previous studies have shown that the abundance of mRNA transcripts is does not completely correlate with expression level of certain genes^53^. Indeed, we also found only partial correlations between the mRNA and protein expression levels of certain genes^54^. Thus, transcriptomic analyses do not fully represent protein expression^55^. As such, analysis of transcriptomic or proteomic data alone is insufficient for accurate characterization of functional mechanisms; only by combining these approaches can we obtain a more comprehensive understanding of the biological functions of organisms.

In conclusion, we identified important hypoxia-adapted genes and proteins in Tibetan pigs, and by combining RNA-seq and iTRAQ data obtained from pig heart tissues, we elucidated the regulatory relationship between DEGs and DEPs. A combination of transcriptomic and proteomic data revealed several key candidate regulators (*A2M*, *PDLIM3*, *CRYAB*, and *ACE*) and pathways (HIF-1 signaling pathway, cardiovascular development, VEGF signaling pathway, and pathways in cancer) that might play high-priority roles in the hypoxic adaptation of Tibetan pigs. These results provide new insights into the molecular mechanisms involved in hypoxia-adaptation regulatory networks, and a greater understanding of human hypoxic diseases. However, further studies are required to confirm the DEGs and DEPs involved in regulating hypoxia identified in this work.

## Materials and Methods

### Sample preparation and extraction

The experimental design comprised four treatment (comparison) groups (*n = *8 per group): Tibetan and Yorkshire pigs raised in the highlands [Linzhi, Tibet, 3000 m above mean sea level (AMSL)] and lowlands (Beijing, China, 100 m AMSL). The animals were slaughtered at the age of six months, and heart tissue samples were collected, immediately frozen in liquid nitrogen, and stored at −80 °C (Table S1). The procedures for animal care were approved by the Animal Welfare Committee of the State Key Laboratory for Agro-biotechnology of the China Agricultural University (Approval number XK257), and all experiments were conducted in accordance with approved relevant guidelines and regulations.

### RNA isolation, library preparation, and sequencing

Total RNA was isolated using Trizol^®^ reagent (Invitrogen, Waltham, MA, USA)^55^. The integrity and concentration and purity of each sample was evaluated via 1% agarose gel electrophoresis and using a NanoDrop^™^ 2000 Biophotometer (Thermo Fisher Scientific, Waltham, MA, USA), respectively. Samples were then reverse transcribed into cDNA using Superscript II reverse transcriptase (Invitrogen) and random hexamer primers. For RNA-seq analyses, RNA pools were created using equal quantities of RNA from four individuals; two biological replicates were included in each group. RNA-seq libraries were constructed according to the manuals provided by Illumina, Inc. (San Diego, CA, USA), and were sequenced using the HiSeq 2000 platform to generate 100-bp paired-end reads. All RNA sequencing data are deposited in the Gene Expression Omnibus under accession number GSE92981.

### Mapping and annotation of sequencing reads

Raw RNA-seq reads were arranged using CLC Genomics Workbench 4.8 software (CLC Bio, Aarhus, Denmark). All reads for which the quality of more than half of the bases was less than 10, as well as all reads that contained more than two Ns or were smaller than 20 bp in length, were eliminated from subsequent analyses. After removal of the adapters, the remaining clean reads were aligned to the whole pig genome (Sscrofa10.2.72) (ftp://ftp.ensembl.org/pub/release-72/fasta/sus_scrofa/dna/Sus_scrofa.Sscrofa10.2.72.dna.toplevel.fa.gz) using TopHat (version 2.0.9) software^56^; two mismatches were allowed per 100-bp read for each alignment. Finally, BAM files generated using SAMtools^57^ were used for subsequent analysis. Quality control and read statistics were determined using FastQC software (http://www.bioinformatics.babraham.ac.uk/projects/fastqc/).

### Quantification and differential gene analysis for RNA-seq

FPKM values obtained using Cufflink software (version 2.1.1) were used as values for normalized gene expression^58^. Differential expression analyses of four comparison groups (TH *vs*. YH, TH *vs*.TL, YH *vs*. YL, and TL *vs*. YL) were performed using the DESeq R package (1.10.1)^59^. The resulting *P*-values were adjusted using the Benjamini-Hochberg method. Results were expressed as the fold change (FC) of the average expression of case groups to that of the respective control group. Among the four comparison groups, DEGs were identified as those genes for which log_2_ (FC) > 1 and *P* < 0.01.

### Protein isolation, enzymolysis, and iTRAQ labeling

Heart tissues were ground into powder in liquid nitrogen using lysis buffer (Roche). The resulting samples were then ultrasonically disrupted for extraction of total protein. After centrifugation at 10,000 × g for 30 min at 4ly, supernatants were collected, and protein concentrations were determined using an enhanced BCA (bicinchoninic acid) Protein Assay Kit (P0010; Beyotime Biotechnologies, Ltd., Beijing, China), according to the manufacturer’s instructions. The protein samples (200 μg) were mixed with dl-dithiothreitol and alkylated with iodoacetamide, and then treated with trypsin overnight at a trypsin-to-protein ratio of 1:100.

Protein peptides (15 μg) from each group were labeled using an 8plex iTRAQ reagent multiplex kit (SCIEX, Framingham, MA, USA). The samples were labeled as 113 (TH1), 114 (TH2), 115 (YH1), 116 (YH2), 117 (TL1), 118 (TL2), 119 (YL1), and 121 (YL2). The labeled samples were pooled and further fractionated offline using an ÄKTA Purifier 100 (GE Healthcare) with a strong cation exchange column (PolySULFOETHYL A^™^; PolyLC Inc., Columbia, MD, USA). The retained peptides were eluted with Buffer A [10 mM KH_2_PO_4_ in 25% ACN (acetonitrile), pH 3.0] and Buffer B (10 mM KH_2_PO_4_ and 500 mM KCl in 25% ACN, pH 3.0) with a flow rate of 1.0 mL/min.

### LC-MS/MS analysis

Eluted fractions were lyophilized using a centrifugal speed vacuum concentrator (CentriVap^®^ Complete Vacuum Concentrator; Labconco, Kansas City, MO, USA) and dissolved in 0.1% formic acid. Equivalent amounts of peptides from each fraction were mixed and then subjected to reversed-phase nanoflow LC-MS/MS analysis using a high-performance liquid chromatography system (EASY-nLC^™^, Thermo Fisher Scientific) connected to a hybrid quadrupole/time of-flight mass spectrometer equipped with a nano-electrospray ion source. The peptides were separated on a C18 analytical reverse-phase column with mixtures of Solution A (0.1% formic acid in water) and Solution B (0.1% formic acid in ACN). A full MS scan was conducted using a Q Exactive^™^ mass spectrometer (Thermo Fisher Scientific).

### Database search and protein identification and quantification

For peptide identification and quantification, MS/MS data were searched against the “Sus_scrofa_35257_20151120_uniprot.fasta” file using Mascot 2.2 and Proteome Discoverer^™^ 1.4 software (Thermo Fisher Scientific). A unique protein with at least two unique peptides that had an FDR <0.01^60^ was used for data analysis. Protein quantification was based on the total intensity of the assigned peptides. The average of eight labeled sample mixes was used as a reference, and was based on the weighted average of the intensity of report ions in each peptide identified. The final protein ratios were normalized to the median average protein content of the 8plex samples. FC > 1.2 or FC < 0.83 was set as the threshold for identifying differentially expressed proteins.

### GO and KEGG enrichment analysis of DEGs and DEPs

DEGs and DEPs were classified by GO and KEGG using DAVID online software (https://david.ncifcrf.gov/)^61^. For these analyses, an official gene symbol for each DEG or DEP was uploaded, and the species with the maximum number of annotations was used. The GO terms used were BP, CC, and MF, and KEGG pathways with corrected *P*-values < 0.05 were considered significantly enriched. In addition, IPA software (http://www.ingenuity.com/; Ingenuity Systems, Redwood City, CA, USA) was used to compare the DEGs among the four treatment groups. Accession numbers for these genes were imported into IPA, and the “Core Analysis” function was used to analyze the genes in the context of networks, biological functions, and canonical pathways. Detailed information concerning IPA analyses was published previously^60, 62, 63^. GO annotation and KEGG pathway analysis of the DEPs was also performed using KOBAS 2.0 (http://kobas.cbi.pku.edu.cn/)^64^ online software.

### Verification of RNA-seq and iTRAQ data

RT-qPCR primers were designed to span the exon-exon boundaries of eight genes selected by RNA-seq; the characteristics of the primers are listed in Table S2. RT-PCR analysis was performed using a SYBR^®^ Green I PCR Master Mix Kit (FP204; Tiangen Biotech Co. Ltd., Beijing, China) on a CFX96^™^ Real-Time PCR Detection System (Bio-Rad, Hercules, CA, USA), according to the manufacturer’s instructions.

The results for TH *vs*. YH obtained by iTRAQ analysis were validated via the label-free technique, as described previously. Label-free quantification is a mass spectrometry method used to determine the relative amounts of proteins of interest in two or more biological samples. Unlike other methods of protein quantification, label-free quantification does not use a stable isotope-containing compound to chemically bind to, and thus, label the protein^63, 65, 66^.

## Electronic supplementary material

Supplementary Figure S1-S9 and Table S1-S3

Supplementary Table S4

Supplementary Table S5

Supplementary Table S6

Supplementary Table S7

Supplementary Table S8

Supplementary Table S9
