# Supplementary material for: Comparative transcriptomic and proteomic analyses provide insights into the key genes involved in high-altitude adaptation in the Tibetan pig
Source: Sci Rep. 2017 Jun 16;7:3654. doi: 10.1038/s41598-017-03976-3 (PMC5473931; doi:10.1038/s41598-017-03976-3)
Supplement: Supplementary file 1 — Supplementary Figure S1-S9 and Table S1-S3 [file 41598_2017_3976_MOESM1_ESM.pdf]

Comparative transcriptomic and proteomic analyses provide insights into the key genes involved in high-altitude adaptation in the Tibetan pig

Bo Zhang[1], Yangzom Chamba[2], Peng Shang[1,2], Zhixiu Wang[1], Jun Ma[1], Liyuang Wang[1], Hao Zhang[1,*]

[1]National Engineering Laboratory for Animal Breeding, China Agricultural University, No. 2 Yuanmingyuan West Rd., Beijing 100193, China

[2] Tibet Agriculture and Animal Husbandry College, Linzhi, Tibet 860000, China

[*]Corresponding author: zhanghao827@163.com.

Supplementary information

Supplementary Figures: 2
Supplementary Figure S1..................2
Supplementary Figure S2..................3
Supplementary Figure S3..................4
Supplementary Figure S4..................5
Supplementary Figure S5..................6
Supplementary Figure S6..................7
Supplementary Figure S7..................8
Supplementary Figure S8..................9
Supplementary Figure S9..................10
Supplementary Tables:..................11
Supplementary Table S1..................11
Supplementary Table S2..................12
Supplementary Table S3..................13
Supplementary Table S4: Please see the Excel file..................14
Supplementary Table S5: Please see the Excel file..................14
Supplementary Table S6: Please see the Excel file..................14
Supplementary Table S7: Please see the Excel file..................14
Supplementary Table S8: Please see the Excel file..................14
Supplementary Table S9: Please see the Excel file..................14

Supplementary Figures:

Supplementary Figure S1

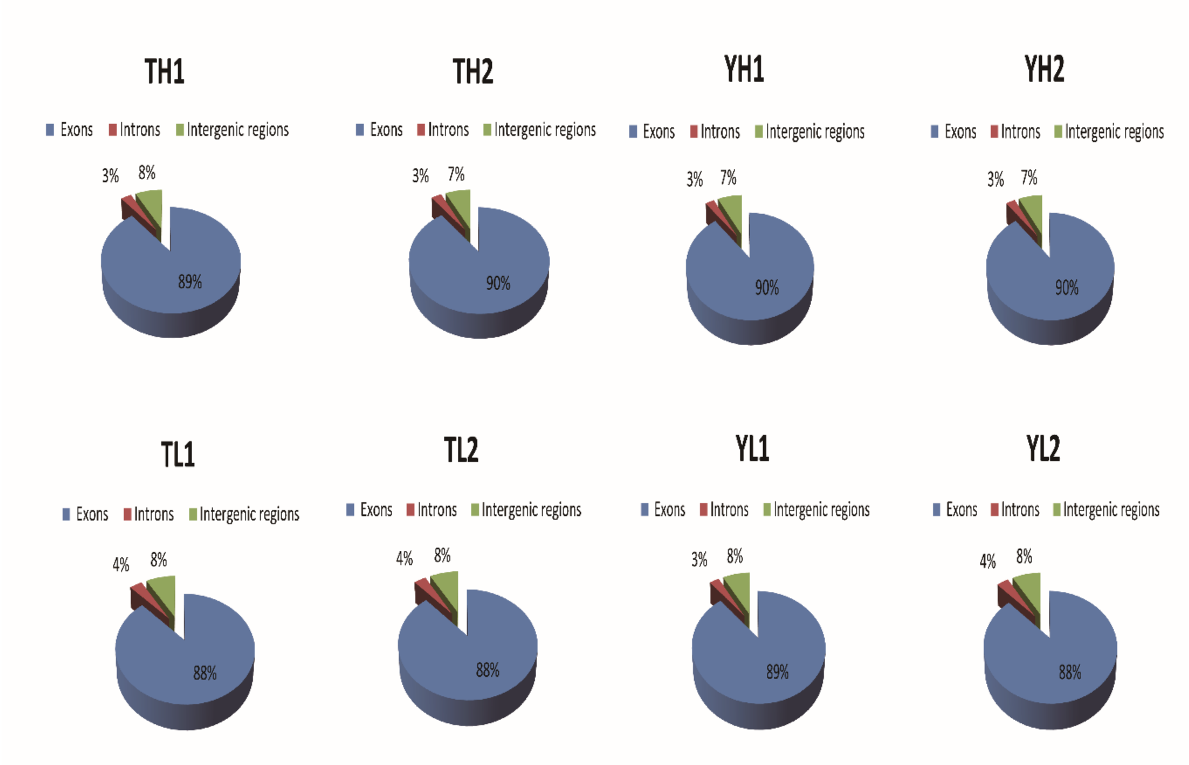

Figure S1. Distribution of reads mapped to the reference genes of eight samples.

Supplementary Figure S2

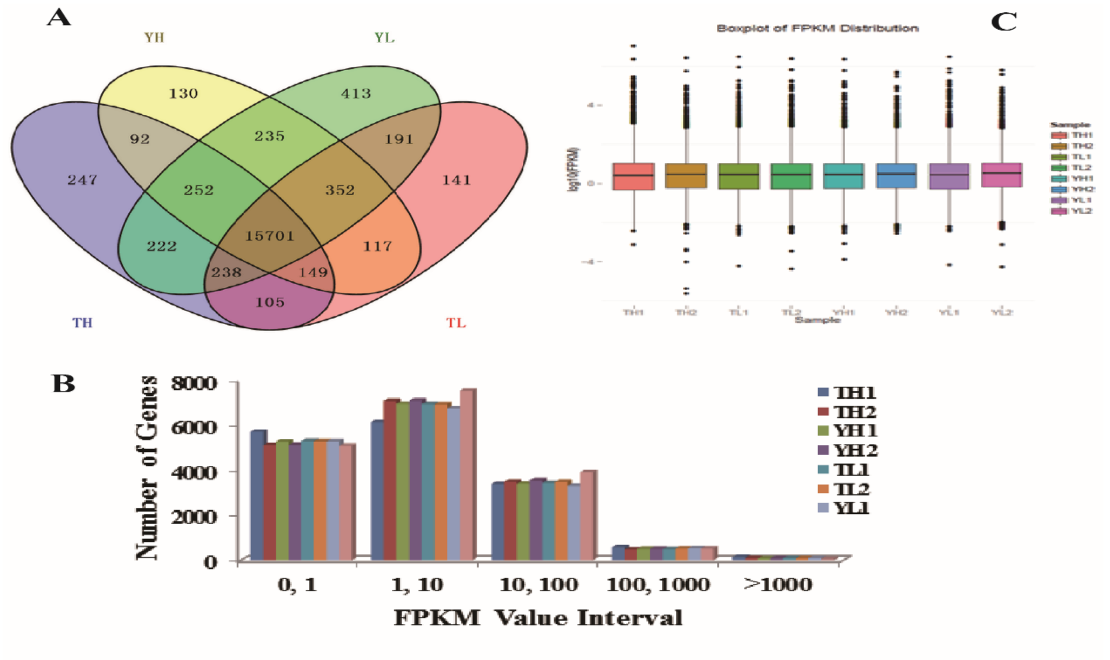

Figure S2. Overview of RNA transcriptome profiles in pig heart tissue. A) Venn diagrams demonstrating relationship between differentially expressed genes (DEGs) in four treatment groups; B) Boxplot of fragments per kilobase of exon per million mapped fragments (FPKM) distributions in eight samples; C) Distributions of expression values of eight samples.

Supplementary Figure S3

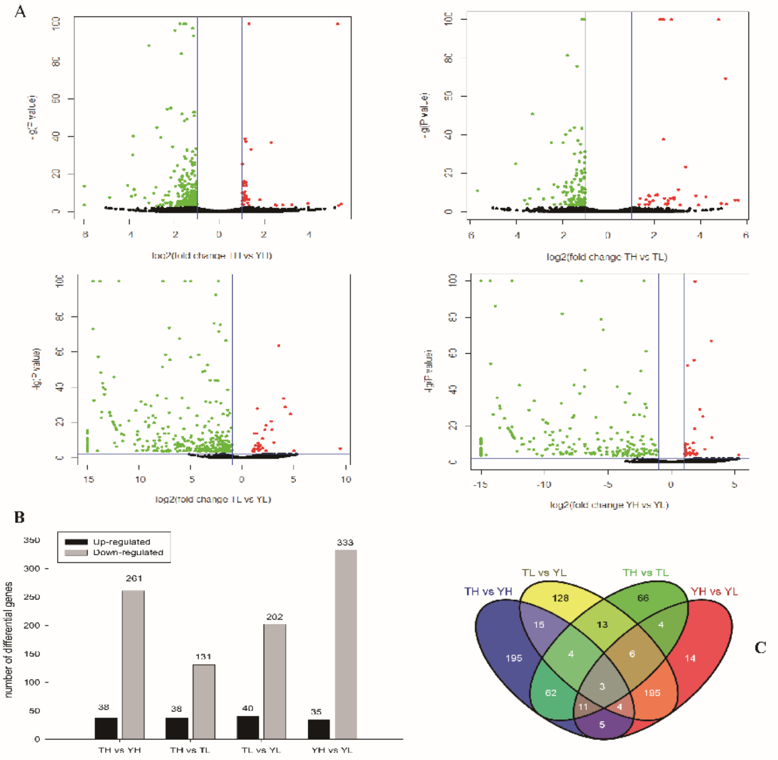

Figure S3. Differentially expressed genes (DEGs) for RNA-seq in four comparison groups. A) Volcano plot displaying DEGs among the four groups. The y-axis corresponds to the mean expression value of $\log_{10}$ (*P*- value), and the x-axis displays the $\log_2$ fold change value. Upregulated and downregulated genes ($P < 0.01$) are shown in red and green, respectively. Black dots represent genes with similar expression levels; B) DEG regulatory trends in the four comparison groups. $FC > 2$ represents upregulated genes and $FC < 1/2$ represents downregulated genes; C) Comparison of DEGs among the four comparison groups using Venn diagrams. Numbers in overlapping areas refer to common DEGs.

Supplementary Figure S4

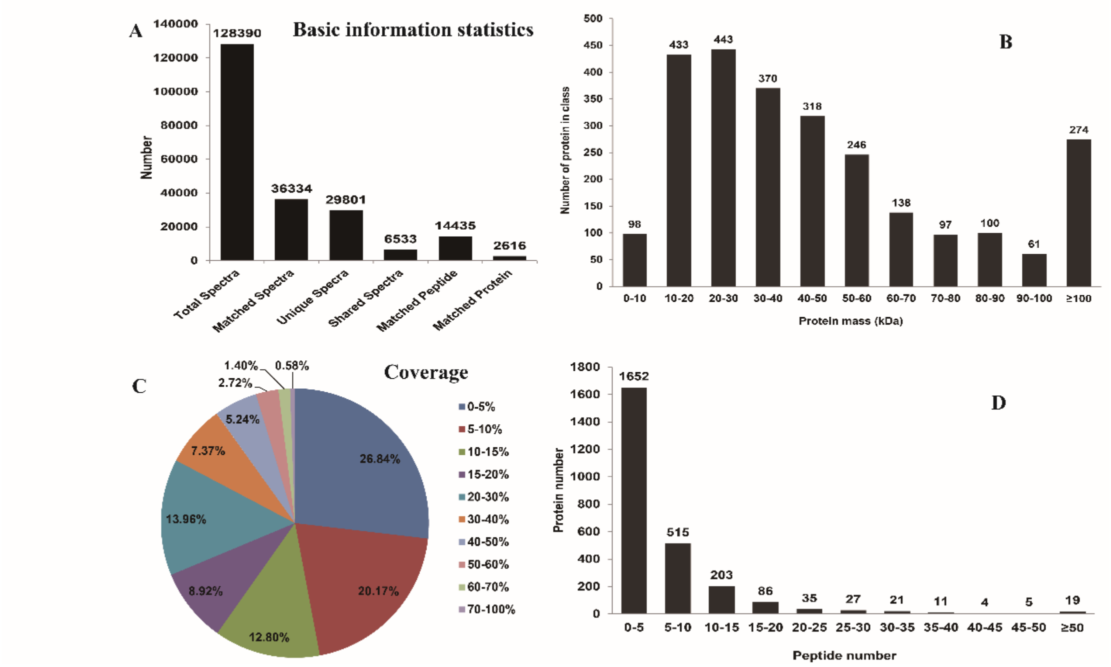

Figure S4. Overview of protein identification information. (a) Basic information on protein identification. (b) Distribution of the proteins identified according to molecular weight. (c) Protein coverage by the peptides identified. (d) Distribution of proteins containing different numbers of identified peptides.

Supplementary Figure S5

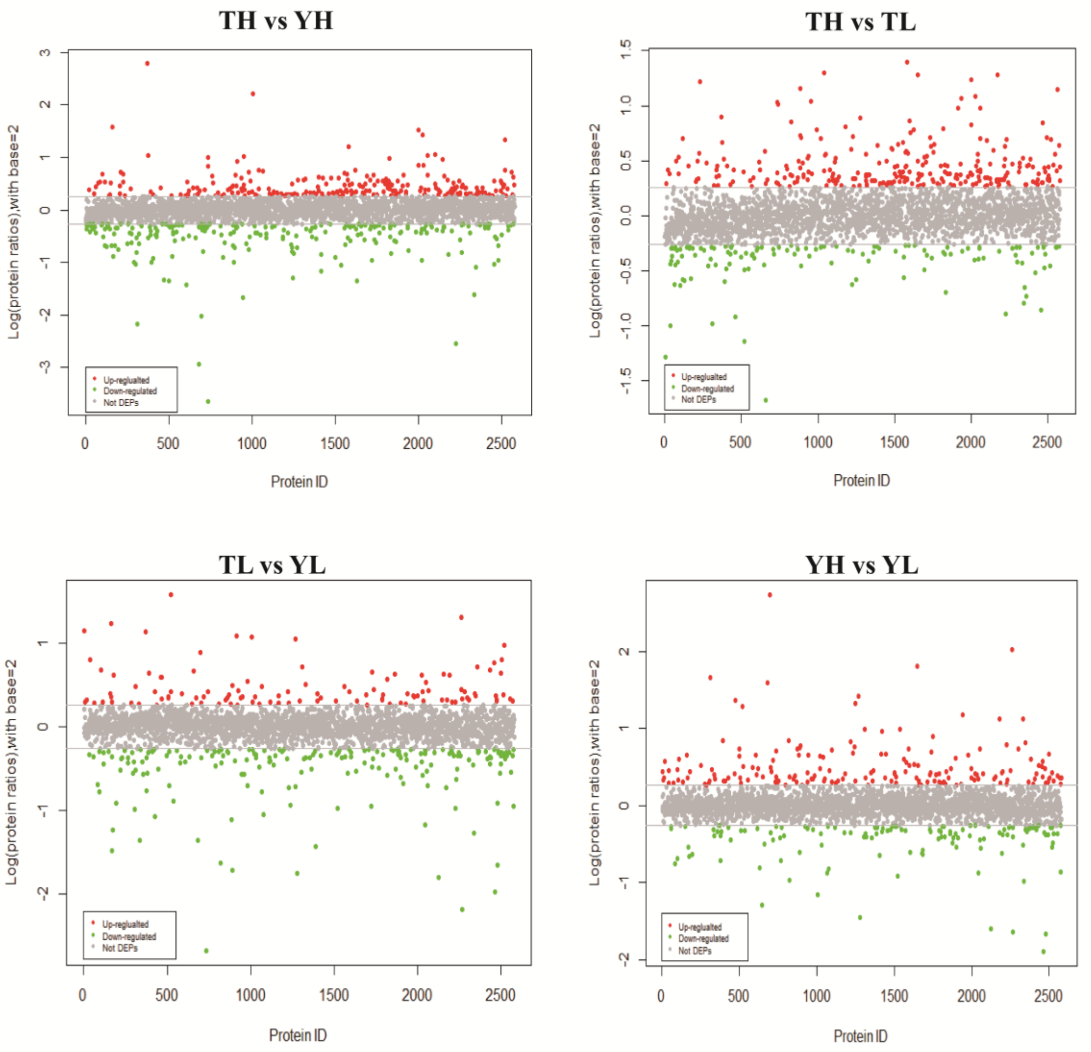

Figure S5. Differentially expressed proteins (DEPs) for iTRAQ in the four comparison groups.

Supplementary Figure S6

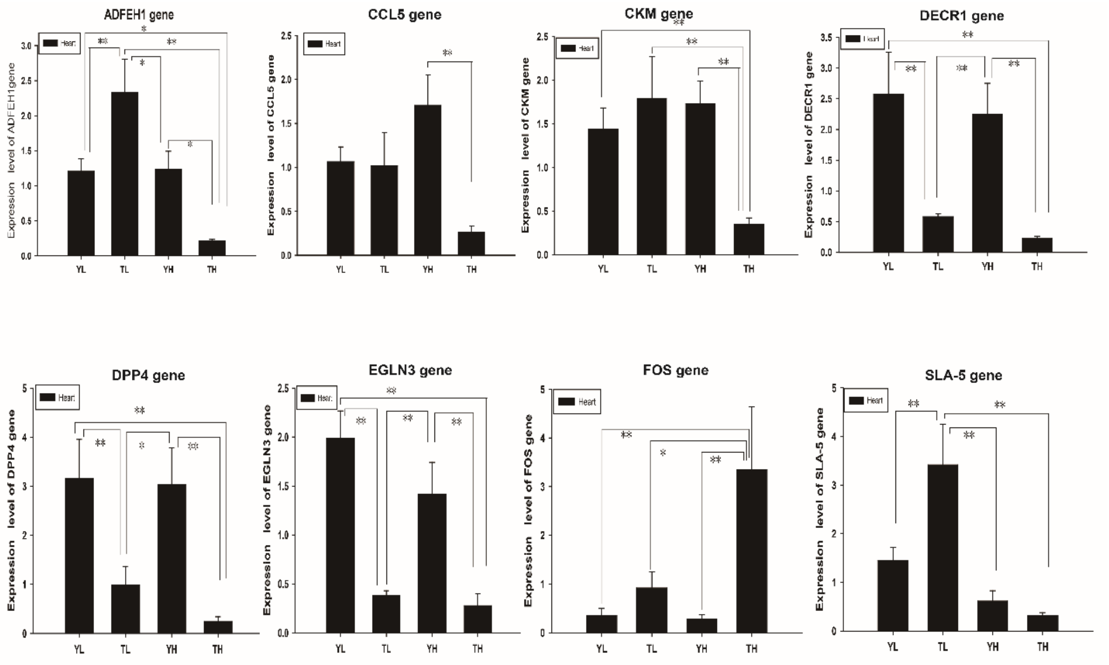

Figure S6. Reverse transcription-quantitative polymerase chain reaction (RT-qPCR) results for eight differentially expressed genes (DEGs). Each bar represents the mean ± S.E. *Significant difference ($P < 0.05$), **Extremely significant difference ($P < 0.01$).

Supplementary Figure S7

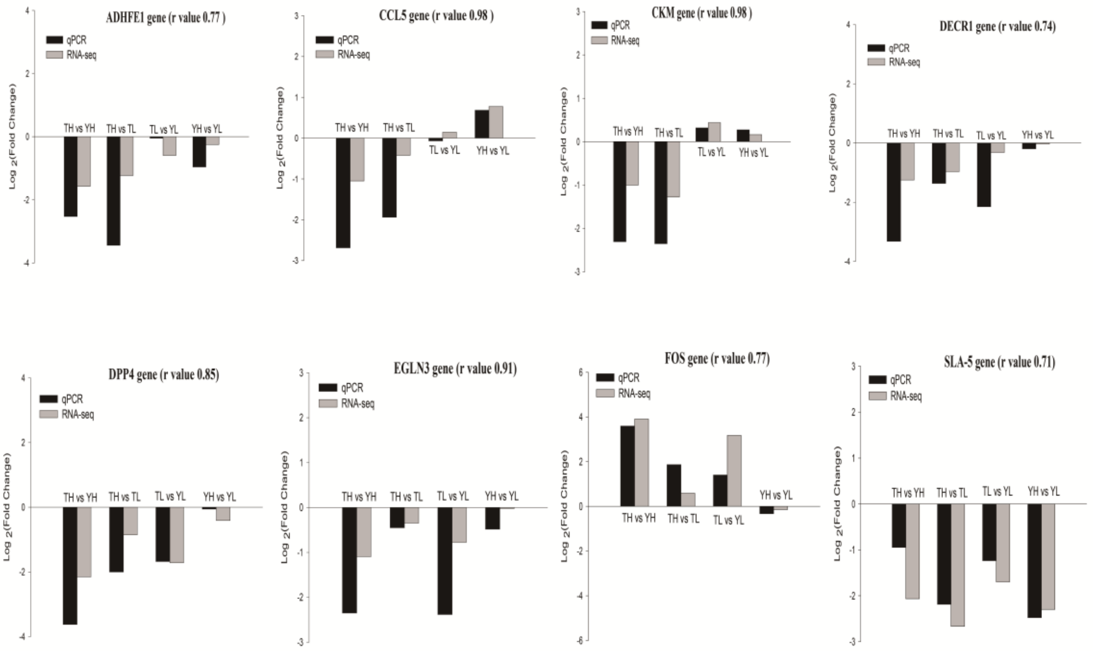

Figure S7. Reverse transcription-quantitative polymerase chain reaction (RT-qPCR) verification of genes in RNA-seq. The expression levels of eight differentially expressed genes (DEGs) were detected by RT-qPCR. r represents the Pearson correlation coefficient. The RT-qPCR provided results that were consistent with the RNA-seq data (r > 0.7).

Supplementary Figure S8

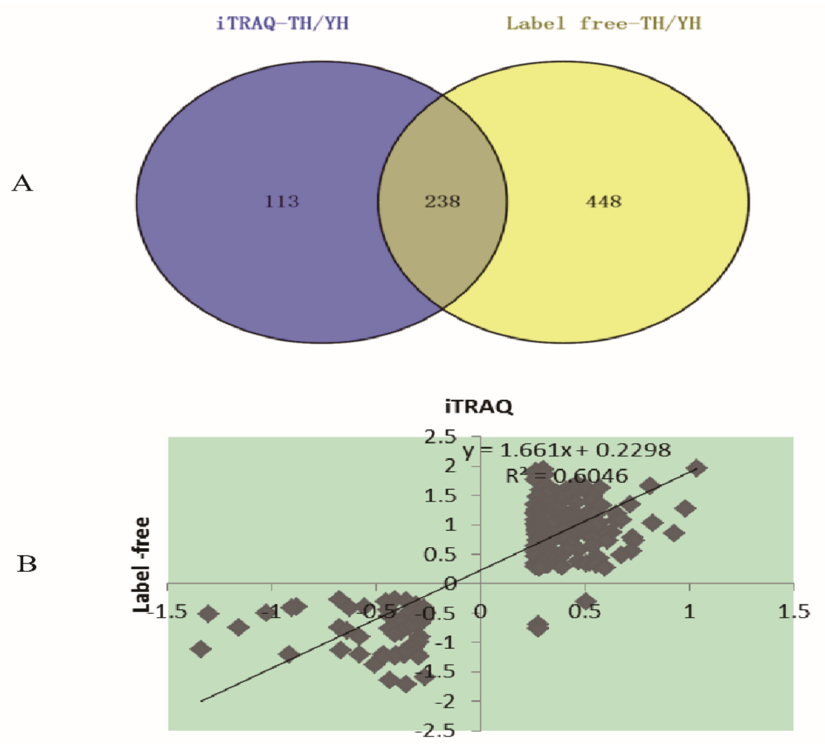

Figure S8. Validation of iTRAQ data by the label-free method. (A) Venn diagram of iTRAQ and label-free for TH *vs.* YH (Tibetan highland pig versus Yorkshire highland pig). (B) Correlation analysis of iTRAQ and label-free.

Supplementary Figure S9

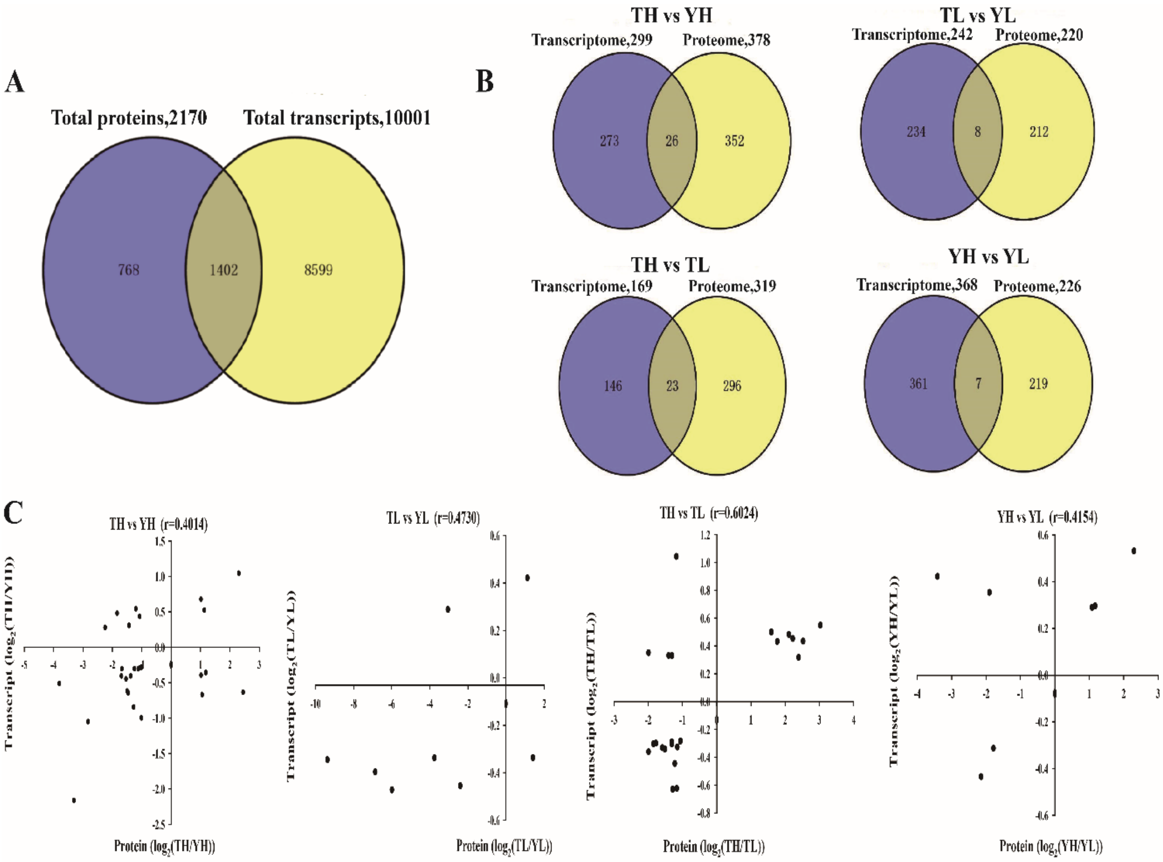

Figure S9. Integrated analysis of differentially expressed genes (DEGs) and differentially expressed proteins (DEPs).

Supplementary Tables:

Supplementary Table S1

Table S1. Sample sets used in the study

Group Name	Sample	Collection Place	Age(months)	Altitude(meter)	Number	Samples in RNA-seq and iTRAQ
Tibet pig in highland (TH)	TH1	Linzhi, Tibet	6	3000	8	mixture of 4 indivduals
	TH2					mixture of 4 indivduals
Tibet pig in lowland (TL)	TL1	Beijing	6	100	8	mixture of 4 indivduals
	TL2					mixture of 4 indivduals
Yorkshire in highland (YH)	YH1	Linzhi, Tibet	6	3000	8	mixture of 4 indivduals
	YH2					mixture of 4 indivduals
Yorkshire in lowland (YL)	YL1	Beijing	6	100	8	mixture of 4 indivduals
	YL2					mixture of 4 indivduals

Supplementary Table S2

Table S2. Primers of eight randomly selected genes for RNA-seq

GeneBank.	Gene	Sequence of primer (5’→3’)
XM_003125603.2	*ADHFE1*	F:GGCTTTGGAAATGCTGGTGT<br>R:GTGAATACTGCTGGGGAGGT
NM_001129946.1	*CCL5*	F:TTATCACCAGAAAGAACCGC<br>R:CTCAAGGCTTCCTCCATC
NM_001129949.1	*CKM*	F:TGTGGGTGAACGAGGAGGA<br>R:AGGTTAGACGGGCAGGTGAG
NM_001190232.2	*DECR1*	F:ATTACAACCATCTATGCCGA<br>R:TTCTTTCTCAAATGCTCCAG
NM_214257.1	*DPP4*	F:TGGAACAGCAGATGATAACG<br>R:GTAGGGTAGAGAGAAGCATTGT
NM_001123113.1	*FOS*	F:TCCCCAGAAGAAGAAGAGAA<br>R:CGCTTGGAGTGTGTCAGTC
NM_001114056.1	*SLA-5*	F:TGGTTCTCGTCCTGGTCGCT<br>R:AAGAAACACATCAGAGCCCT
XM_001928507.4	*EGLN3*	F:GGGAAATCATTCATAGCAGA<br>R:TGAGGGCAGGCTCGGTTTT
AJ312193	*β-actin*	F:TCTGGCACCACACCTTCTA<br>R:AAGGTCTCGAACATGATCTG
NM_001206359	*GAPDH*	F:GGTCACCAGGGCTGCTTTTA<br>R:CCTTGACTGTGCCGTGGAAT

Note: All primers were designed based on mRNA sequences. GAPDH and β-actin genes were used as internal controls of genes.

Supplementary Table S3

Table S3. Summary of the sequencing reads alignment to the pig reference genome

Sample	TH1	TH2	YH1	YH2	TL1	TL2	YL1	YL2
Clean reads	86,923,902	52,533,158	65,184,090	67,468,286	64,210,492	79,379,614	62,073,534	73,689,732
Total mapped reads	72,420,772 (83.32%)	43,113,696 (82.07%)	55,389,296 (84.97%)	57,467,339 (85.18%)	52,884,724 (82.36%)	66,027,615 (83.18%)	52,757,743 (84.99%)	61,437,704 (83.37%)
Unique mapped reads	66,820,768 (76.87%)	39,327,822 (74.86%)	50,609,288 (77.64%)	51,552,262 (76.41%)	48,132,537 (74.96%)	60,045,081 (75.64%)	48,257,243 (77.74)	55,965,043 (75.95%)
Multiple mapped reads	5,600,004 (6.44%)	3,785,874 (7.21%)	4,780,008 (7.33%)	5,915,077 (8.77%)	4,752,187 (7.40%)	5,982,534 (7.54%)	4,500,500 (7.25%)	5,472,661 (7.43%)
Fragments mapped to Exons	31,537,503 (43.55%)	18,889,684 (43.81%)	23,625,490 (42.65%)	24,270,894 (42.23%)	22,279,021 (42.13%)	27,850,601 (42.18%)	22,295,735 (42.26%)	25,385,755 (41.32%)
Fragments mapped to Introns	2,230,119 (3.08%)	1,288,024 (2.99%)	1,521,350 (2.75%)	1,613,847 (2.81%)	1,893,572 (3.58%)	2,252,673 (3.41%)	1,485,644 (2.82%)	2,192,840 (3.57%)
Fragments mapped to Intergenic	5,564,427 (7.68%)	3,149,067 (7.30%)	4,133,613 (7.46%)	4,113,394 (7.16%)	4,415,997 (8.35%)	5,363,928 (8.12%)	4,215,620 (7.99%)	5,248,920 (8.54%)
Reads overlapped with exons	33,088,723 (45.69%)	19,786,921 (45.89%)	26,108,843 (47.14%)	27,469,204 (47.80%)	24,296,134 (45.94%)	30,560,413 (46.28%)	24,760,744 (46.93%)	28,610,189 (46.57%)
Total unmapped reads	14,503,130 (16.68%)	9,419,462 (17.93%)	9,794,794 (15.03%)	10,000,947 (14.82%)	11,325,768 (17.64%)	13,351,999 (16.82%)	9,315,791 (15.01%)	12,252,028 (16.63%)
Genes with >1000 FPKM	141 (0.88%)	95 (0.58%)	81 (0.50%)	89 (0.54%)	96 (0.59%)	90 (0.55%)	98 (0.61%)	64 (0.37%)
Genes with 100 to 1000 FPKM	577 (3.62%)	481 (2.96%)	504 (3.11%)	507 (3.10%)	488 (3.00%)	514 (3.15%)	528 (3.31%)	521 (3.05%)
Genes with 10 to 100 FPKM	3,387 (21.23%)	3,485 (21.44%)	3,403 (20.99%)	3,548 (21.69%)	3,432 (21.11%)	3,483 (21.37%)	3,306 (20.71%)	3915 (22.88%)
Genes with 1 to 10 FPKM	6,141 (38.49%)	7,082 (43.57%)	6,954 (42.90%)	7,096 (43.38%)	6,946 (42.73%)	6,932 (42.52%)	6,748 (42.28%)	7526 (43.99%)
Genes with <1 FPKM	5,709 (35.78%)	5,111 (31.44%)	5,268 (32.50%)	5,116 (31.28%)	5,295 (32.57%)	5,283 (32.41%)	5,282 (33.09%)	5083 (29.71%)
Total genes	15,955	16,254	16,210	16,356	16,257	16,302	15,962	17,109

Supplementary Table S4: Please see the Excel file

Table S4. All differentially expressed genes (DEGs) from heart tissues for RNA-seq.

Supplementary Table S5: Please see the Excel file

Table S5. Functional analysis of differentially expressed genes (DEGs) using DAVID.

Supplementary Table S6: Please see the Excel file

Table S6. Functional analysis of differentially expressed genes (DEGs) using IPA.

Supplementary Table S7: Please see the Excel file

Table S7. All differentially expressed proteins (DEPs) from heart tissues for iTRAQ.

Supplementary Table S8: Please see the Excel file

Table S8. Overlapping proteins in the comparison groups.

Supplementary Table S9: Please see the Excel file

Table S9. Gene Ontology (GO) functional analysis of differentially expressed proteins (DEPs).
